# Antibiotic-Associated Overanticoagulation Unmasking Prosthetic Valve Endocarditis Diagnosed by Fluorodeoxyglucose-Positron Emission Tomography/Computed Tomography (FDG-PET/CT)

**DOI:** 10.7759/cureus.106816

**Published:** 2026-04-10

**Authors:** José A Sánchez Aguirre, Gladis Faustino Maravilla, Ana K Garro Almendaro, Diana L Ortega García, Carlos R Zamora Alemán

**Affiliations:** 1 Internal Medicine, Centro Médico Nacional 20 de Noviembre, Instituto de Seguridad y Servicios Sociales de los Trabajadores del Estado (ISSSTE), Mexico City, MEX; 2 Cardiology, Centro Médico Nacional 20 de Noviembre, Instituto de Seguridad y Servicios Sociales de los Trabajadores del Estado (ISSSTE), Mexico City, MEX; 3 Radiology, Hospital Universitario “Dr. José Eleuterio González,” Universidad Autónoma de Nuevo León, Monterrey, MEX

**Keywords:** enterococcus faecalis, fdg-pet/ct, overanticoagulation, prosthetic valve endocarditis, vitamin k antagonists

## Abstract

Vitamin K antagonists (VKAs) remain the standard anticoagulation therapy in patients undergoing mechanical valve replacement; despite their efficiency in this clinical context, they are associated with a very narrow therapeutic window, a factor that links them to a major risk of bleeding. Their broad involvement in the coagulation cascade and their interactions with multiple commonly used drugs can lead to supratherapeutic anticoagulation. Furthermore, prosthetic valve infective endocarditis is a serious complication that poses a diagnostic challenge, especially when echocardiographic findings are inconclusive.

We present the case of a 50-year-old man with a mechanical aortic valve prosthesis secondary to severe aortic stenosis, on anticoagulation therapy with acenocoumarol, who presented with severe over-anticoagulation, with multiple hemorrhagic complications, including hemothorax, chronic subdural hematoma, and extensive intramuscular hematomas involving the iliopsoas muscle and deep compartments of the thigh. The onset of these complications was temporally associated with the administration of an antibiotic regimen containing amoxicillin, secondary to an upper respiratory tract infection. During his hospital stay, persistent fever and the isolation of *Enterococcus faecalis* from central and peripheral blood cultures raised suspicion of probable infective endocarditis; however, both transthoracic and transesophageal echocardiograms were inconclusive for vegetations. Therefore, positron emission tomography (PET) with fluorodeoxyglucose (FDG) combined with computed tomography (CT) was performed, revealing focal hypermetabolic uptake around the prosthetic aortic valve annulus, consistent with prosthetic valve endocarditis; targeted antimicrobial therapy was initiated, and the patient was subsequently referred for surgical valve replacement, which was performed after the completion of antibiotic treatment with satisfactory clinical progress.

This case highlights the vast clinical consequences of anticoagulation instability associated with VKAs, and especially the exponential increase in risk due to drug interactions, as in this case, linked to antibiotic therapy. Furthermore, it emphasizes the value of FDG-PET/CT in the diagnosis of prosthetic valve endocarditis, particularly when echocardiographic findings are inconclusive.

## Introduction

Despite remaining the primary anticoagulant therapy for patients with mechanical heart valve prostheses, the narrow therapeutic index and variability in the international normalized ratio (INR) control with vitamin K antagonists (VKAs) make them difficult to manage, associated with a high risk of bleeding complications; therefore, they require careful monitoring to maintain anticoagulation within the desired therapeutic range [1].

Fluctuations in anticoagulation control with VKAs are frequently associated with drug interactions, dietary variations, or acute illnesses. Precisely, drug interactions are one of the most frequent causes of supratherapeutic elevations that lead to over-anticoagulation. Antibiotics are particularly relevant due to their association with alterations in the gut microbiota, which participates in vitamin K synthesis, and because they directly or indirectly interfere with the hepatic metabolism of anticoagulants [2-4]. Infective endocarditis affecting prosthetic valves represents a considerable proportion of all endocarditis cases, and its association with increased morbidity and mortality makes timely diagnosis imperative.

Despite numerous advances in cardiac imaging, diagnosis remains a challenge because prosthetic material often hinders visualization of vegetations, especially in echocardiographic evaluation. In recent years, fluorodeoxyglucose positron emission tomography combined with computed tomography (FDG-PET/CT) has emerged as a useful imaging modality for detecting inflammatory activity around prosthetic cardiac devices [5].

This report describes a patient who developed severe over-anticoagulation with multiple hemorrhagic complications following antibiotic exposure and was subsequently diagnosed with prosthetic valve endocarditis despite negative echocardiographic findings.

## Case presentation

A 50-year-old man presented to the emergency department with fever, dyspnea, and dark stools. His medical history included a mechanical aortic valve replacement performed four years prior for severe aortic stenosis, chronic atrial fibrillation, and a previous ischemic stroke with hemorrhagic transformation that required decompressive craniectomy; the patient was receiving chronic anticoagulation with acenocoumarol at a dose of 3 mg daily.

Two weeks prior to hospital admission, the patient experienced symptoms consistent with an upper respiratory tract infection, characterized by odynophagia and rhinorrhea; he was treated empirically with amoxicillin 500 mg every 12 hours for seven days, after which the symptoms resolved.

One week before hospitalization, he suffered a fall from standing height, sustaining the impact to the right side of his body and the right temporofrontal region. Three days later, he developed a fever of up to 38.5°C accompanied by progressive dyspnea; outpatient laboratory tests revealed severe anemia and a supratherapeutic INR, so he was immediately referred to the hospital. Upon arrival, he was hemodynamically unstable, with a blood pressure of 70/40 mmHg, non-perfusing mean arterial pressures, capillary refill greater than three seconds, a heart rate of 126 beats per minute, a respiratory rate of 35 breaths per minute, oxygen saturation by pulse oximetry of 84% on room air, and a temperature of 38.3°C; physical examination revealed pallor and evidence of upper gastrointestinal bleeding with melena, and laboratory studies upon admission revealed a markedly elevated INR of 6.4, along with very low levels of hemoglobin to 5.4 g/dL, leukocytosis of 29,700/µL due to neutrophilia, with a normal platelet count of 354,000/µL (Table 1).

**Table 1:** Laboratory findings on admission Laboratory findings on hospital admission and reference ranges. INR: international normalized ratio

Parameter	Value	Reference Range
Hemoglobin	5.4 g/dL	13.5-17.5 g/dL
Leukocytes	29,700/µL	4,000-10,000/µL
Platelets	354,000/µL	150,000-450,000/µL
INR	6.4	0.8-1.2

**Figure 1:**
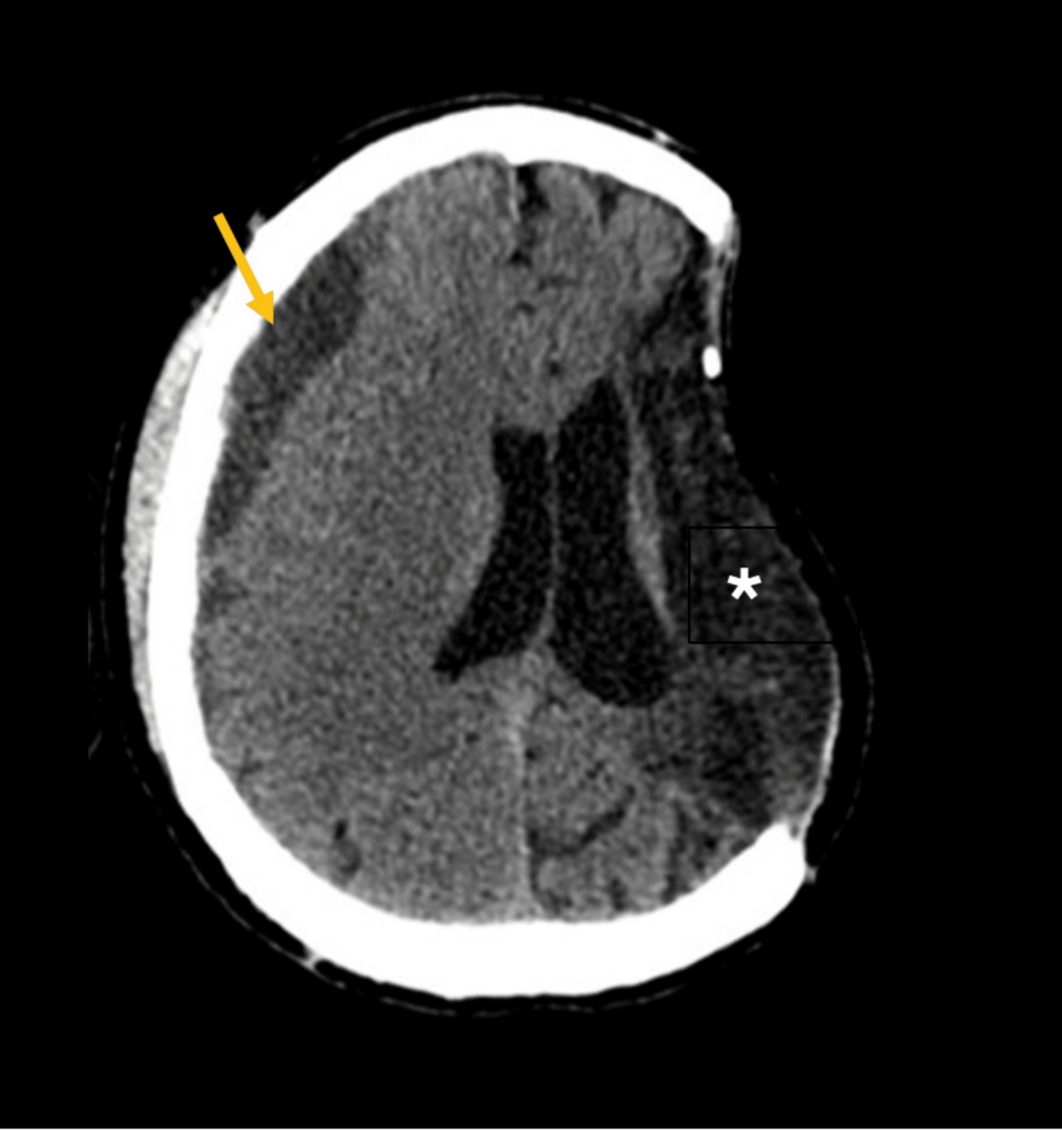
Non-contrast cranial computed tomography Axial non-contrast head CT demonstrates postoperative changes related to prior left-sided decompressive craniectomy (asterisk) with a right frontoparietal chronic subdural hematoma (arrow).

Subsequently, the patient was resuscitated with intravenous fluids and transfusion of three units of packed red blood cells, as well as five units of fresh frozen plasma, which reduced the INR to below 2. Aerobic and anaerobic peripheral blood cultures were obtained before initiating antimicrobial therapy, and multiple imaging studies were ordered. The non-contrast cranial CT scan showed post-surgical changes related to the previous craniectomy and a chronic right frontoparietal subdural hematoma, without evidence of active intracranial bleeding (Figure 1).

**Figure 2:**
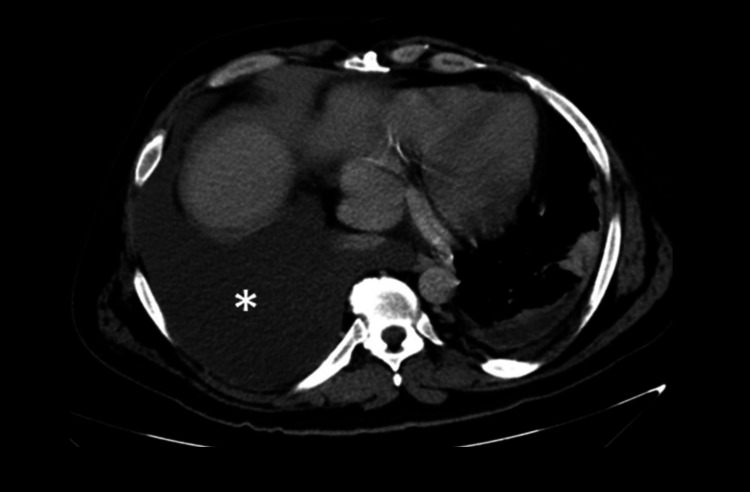
Contrast-enhanced chest computed tomography Axial contrast-enhanced CT of the chest demonstrating a large right-sided pleural collection with high attenuation (asterisk), compatible with hemothorax, occupying a substantial portion of the right hemithorax and causing compressive atelectasis of the adjacent lung parenchyma.

**Figure 3:**
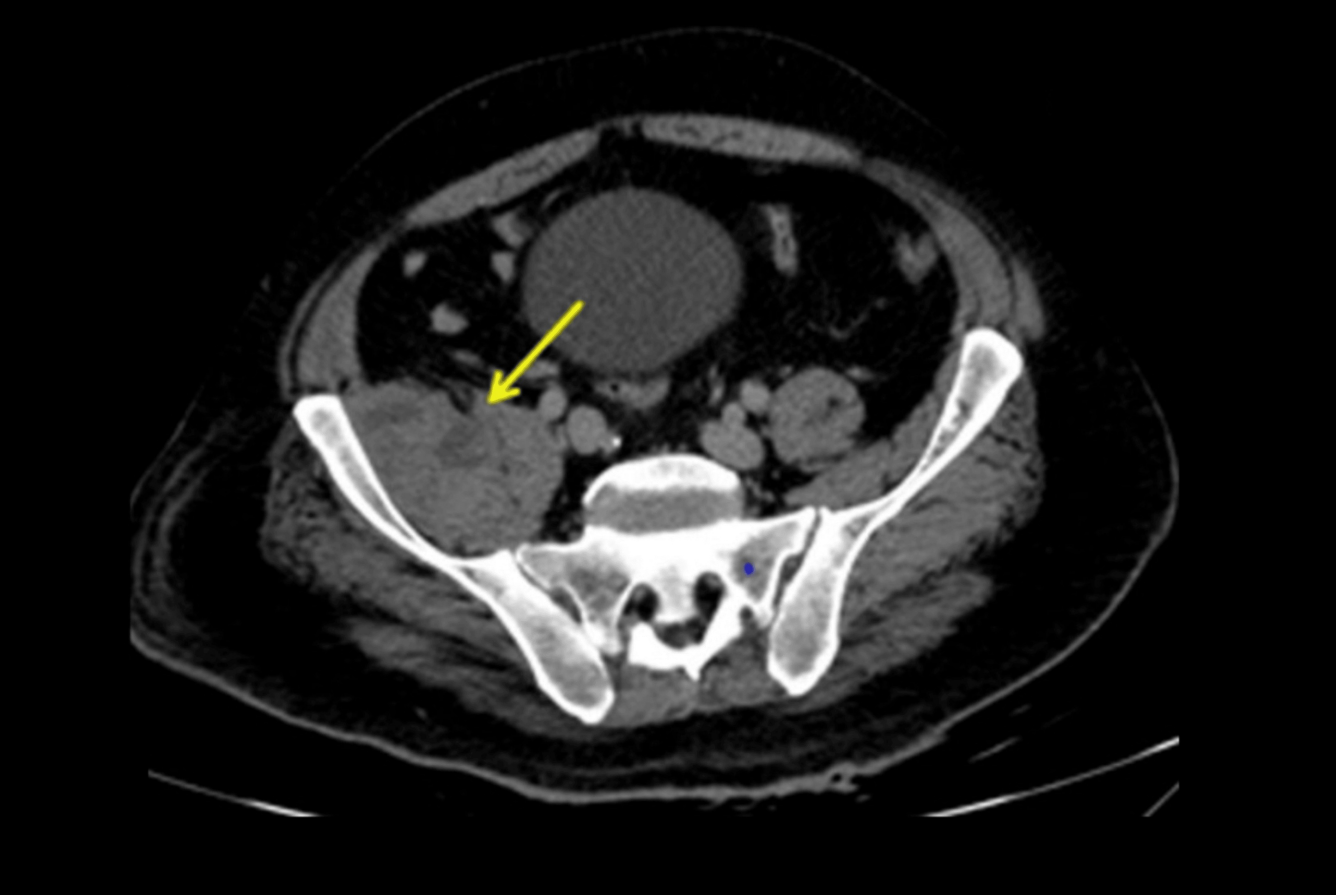
Contrast-enhanced abdominal computed tomography Axial contrast-enhanced CT demonstrating enlargement of the right iliopsoas muscle with a heterogeneous intramuscular collection (arrow) consistent with an iliopsoas hematoma.

However, the contrast-enhanced chest CT scan revealed a large, high-attenuation right pleural collection, consistent with hemothorax, occupying approximately 50% of the right hemithorax, which was causing compressive atelectasis of the adjacent lung (Figure 2). The abdominal CT scan also showed an enlargement of the right iliopsoas muscle with a heterogeneous intramuscular collection consistent with an iliopsoas hematoma (Figure 3).

Following these findings, an endopleural drain was placed, and, once the clinical stability of the patient allowed it, the intramuscular collections were drained under fluoroscopic guidance. Cultures obtained from these sites showed no bacterial growth; however, at the beginning of hospitalization, broad-spectrum empirical antibiotic treatment was initiated, the fever disappeared 96 hours after admission and 48 hours after drainage of the intramuscular collections.

**Figure 4:**
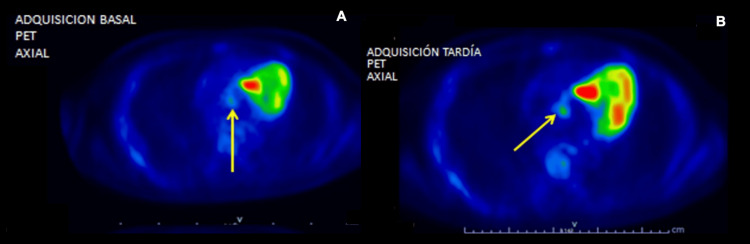
FDG-PET/CT imaging (A) Axial baseline FDG-PET acquisition demonstrating focal hypermetabolic uptake adjacent to the prosthetic aortic valve annulus, adjacent to the mitral-aortic junction (arrow), with a maximum standardized uptake value (SUVmax) of 3.8. (B) Delayed acquisition showing persistent focal FDG uptake in the same region, with an SUVmax of 4.4, supporting inflammatory activity (arrow). FDG: fluorodeoxyglucose; PET/CT: positron emission tomography/computed tomography

Initial blood cultures yielded growth of *Enterococcus faecalis*, sensitive to ampicillin; therefore, due to the fever, leukocytosis with neutrophil predominance, the direct risk factor of having a prosthetic valve, and the bacteremia, further diagnostic workup was conducted to rule out infective endocarditis. A transthoracic echocardiogram showed prosthetic valve dysfunction with severe aortic regurgitation; however, there was no evidence of vegetations. The transesophageal echocardiogram also failed to identify definitive vegetations; however, a small, rounded, and hyperechogenic structure was observed adjacent to the prosthetic valve annulus, the nature of which could not be clearly characterized due to its size and characteristics. Although the initial clinical suspicion for infective endocarditis was low, the inability to reliably distinguish between vegetation and thrombus in the previous image, along with persistent bacteremia without clearance in serial blood cultures, prompted further evaluation. An FDG-PET/CT scan was performed, revealing a linear hypermetabolic focus surrounding the prosthetic aortic valve annulus, adjacent to the mitral-aortic junction, with a maximum standardized uptake value (SUVmax) of 3.8 on initial acquisition and 4.4 on delayed imaging. These findings were consistent with prosthetic valve infection (Figure 4). Furthermore, based on clinical, microbiological, and imaging findings, the case fulfilled the criteria for definite infective endocarditis according to the modified Duke criteria.

After stabilization and adequate clinical progress, the patient was discharged; considering its oral bioavailability and feasibility for outpatient treatment, linezolid was selected as antimicrobial therapy for four weeks; after assessment and balancing the high thrombotic risk associated with mechanical valve prosthesis against the recent hemorrhagic complications, anticoagulation was cautiously restarted, reintroducing acenocoumarol 14 days after the last recorded bleeding event. After completing antibiotic therapy, the patient was referred for evaluation by the cardiothoracic surgery service, and the replacement of the dysfunctional prosthetic valve was successfully performed.

## Discussion

VKAs remain the cornerstone of anticoagulant therapy in patients with mechanical prosthetic heart valves, given their proven efficacy in preventing thrombotic complications. However, their pharmacodynamic and pharmacokinetic characteristics result in a very narrow therapeutic index, with significant intra- and inter-patient variability, leading to a high risk of bleeding when the INR exceeds the ideal therapeutic range. Major bleeding complications occur in approximately 2-5% of patients on long-term oral anticoagulant therapy each year, making it the most significant adverse effect associated with VKA use [1]. In addition to the inherent complexity of managing VKAs, multiple intrinsic and extrinsic factors can alter their metabolism; drug interactions are one of the most frequent causes of INR instability. Numerous medications can potentiate the anticoagulant effect by altering hepatic metabolism, interfering with plasma protein binding, or modifying vitamin K bioavailability. Antibiotics represent a group of medications with a significant association, primarily due to their harmful effects on the gut microbiota, which plays an essential role in vitamin K synthesis. Recent observational studies have linked the concomitant administration of coumarins with various antibiotic regimens to a significant increase in the risk of bleeding [2-4]. Due to its very limited metabolic interactions, amoxicillin is considered a relatively safe drug; however, its combination with the potentiation of the anticoagulant effect of coumarins can lead to hemorrhagic complications.

Severe over-anticoagulation can cause a wide spectrum of hemorrhagic manifestations. Intracranial hemorrhage remains the most severe complication due to its high morbidity and mortality [6]. Other anatomical sites frequently associated with hemorrhagic manifestations include gastrointestinal bleeding, hemothorax, retroperitoneal hemorrhage, and extensive intramuscular hematomas. Iliopsoas muscle hematoma is a particularly well-known complication in anticoagulated patients and can manifest as anemia, hemodynamic instability, or femoral neuropathy. In the present case, bleeding sites were identified at virtually all levels, except for intracranial hemorrhage. Although hematomas are usually sterile collections of blood, they can sometimes become secondarily infected, especially in the presence of bacteremia; devitalized tissue and a diminished local immune response can promote bacterial proliferation within these cavities. Infected hematomas have been described in association with various pathogens, including *Staphylococcus aureus*, enterococci, and Gram-negative organisms; however, direct progression from an infected hematoma to infective endocarditis is relatively uncommon, and more frequently, bacteremia originates from other primary infectious sources. In the International Collaboration on Endocarditis (ICE) study cohort, skin and soft tissue infections accounted for only 4% of the primary foci identified in patients with infective endocarditis [7]. Although a potential relationship may be considered, no direct causal link can be established in this case.

Infective endocarditis of prosthetic valves accounts for approximately 20% of all endocarditis cases and occurs in 1-6% of patients with prosthetic valves during their lifetime [8]. Prosthetic valve endocarditis is associated with higher morbidity and mortality compared to native valve infections due to diagnostic challenges and a higher frequency of complications [9]. The timing of endocarditis in relation to valve implantation is usually associated with the spectrum of suspected pathogens. Early infections are often associated with the surgical procedure or hospital stay, and the most frequent pathogens are *S. aureus* or coagulase-negative staphylococci. Late infections, on the other hand, tend to resemble native valve infections and frequently involve streptococci and enterococci [7].

*E. faecalis* has emerged as an increasingly common cause of infective endocarditis, especially in elderly patients and those with comorbidities. In a prospective study, infective endocarditis developed in up to 26% of patients with *E. faecalis* bacteremia, highlighting the importance of systematic evaluation upon isolation of this pathogen [10].

The availability and non-invasive nature of transthoracic echocardiography (TTE) continue to make it the initial diagnostic approach; however, its diagnostic performance is highly variable, and its sensitivity in evaluating native valves for the detection of vegetations ranges from 60% to 70% [11,12]. Transesophageal echocardiography (TEE) has a significantly higher sensitivity, between 90% and 100%, for the detection of vegetations, abscesses, and perivalvular complications [11-13]; however, in the context of suspected prosthetic valve infection, the diagnostic performance of both modalities can be significantly reduced due to multiple acoustic artifacts generated by the material, in addition to the higher frequency of complications located in the perivalvular region. Because of this, the sensitivity of TTE can decrease to 40-50%, while TEE maintains a higher sensitivity, generally between 80% and 90% [11-13]. These diagnostic limitations explain why, in patients with high clinical suspicion and negative or inconclusive echocardiographic studies, current guidelines recommend considering advanced imaging techniques, such as FDG-PET/CT, particularly in cases of prosthetic valve endocarditis [13].

FDG-PET/CT detects metabolic activity associated with inflammatory processes and can identify infections in the prosthetic material or surrounding tissues [12]. In previous observational studies, which included patients with suspected infective endocarditis, FDG-PET/CT showed an overall sensitivity of approximately 86% in prosthetic valve endocarditis, and a reported specificity approaching 80-85% in prosthetic valve endocarditis. Therefore, incorporating FDG-PET/CT into the diagnostic algorithm can substantially improve the detection of probable prosthetic material infection, even increasing sensitivity compared to the modified Duke criteria, especially in clinical scenarios where echocardiography is negative or inconclusive, but a high clinical suspicion persists [12-14]. Due to these findings, current international guidelines recognize abnormal FDG uptake around prosthetic valves as a major imaging criterion for the diagnosis of infective endocarditis in appropriate clinical contexts [5].

The management of complex cases such as the one presented requires a multidisciplinary approach. Close collaboration between cardiology, infectious diseases, and internal medicine teams is essential to ensure appropriate diagnostic evaluation, targeted antimicrobial therapy, and safe anticoagulation management. In addition, the involvement of clinical pharmacy can play a key role in identifying potential drug-drug interactions and minimizing the risk of adverse events, particularly in patients receiving VKAs. Such coordinated care is critical to optimize clinical outcomes in patients with overlapping infectious and hemorrhagic complications.

## Conclusions

This case involves the relationship between VKAs and the multiple associated drug interactions that complicate their management, as well as dose titration, increasing the risk of over-anticoagulation and, subsequently, the possibility of severe hemorrhagic complications that obscure the patient's prognosis. It reminds us of the imperative need for a detailed evaluation of each patient in this clinical context before prescribing or modifying any medication within their regimen, even in the case of drugs with a traditionally low-risk safety profile, such as amoxicillin.

The limitations of conventional imaging methods became evident during this diagnostic workup, reinforcing the need for advanced imaging techniques in specific clinical settings. FDG-PET/CT has established itself as an important diagnostic modality, capable of identifying periprosthetic inflammatory processes and supporting the diagnosis of infective endocarditis, especially in patients with mechanical valves, in whom inconclusive echocardiographic findings are more common.
